# Reliability and validity of a new HIV-specific questionnaire with adults living with HIV in Canada and Ireland: the HIV Disability Questionnaire (HDQ)

**DOI:** 10.1186/s12955-015-0310-9

**Published:** 2015-08-12

**Authors:** Kelly K. O’Brien, Patricia Solomon, Colm Bergin, Siobhán O’Dea, Paul Stratford, Nkem Iku, Ahmed M. Bayoumi

**Affiliations:** Department of Physical Therapy, University of Toronto, 500 University Avenue, Room 160, Toronto, ON M5G 1V7 Canada; Institute of Health Policy, Management and Evaluation (IHPME), University of Toronto, 4th Floor, 155 College Street, Toronto, ON M5T 3M6 Canada; School of Rehabilitation Science, McMaster University, 1400 Main Street West, Room 408, Hamilton, ON L8S 1C7 Canada; Department of Genito Urinary Medicine and Infectious Diseases, Hospital 5, St. James’s Hospital, James’s Street, Dublin 8, Ireland; Department of Clinical Medicine, Trinity College Dublin, Dublin, Ireland; Centre for Research on Inner City Health, Keenan Research Centre of the Li Ka Shing Knowledge Institute, St. Michael’s Hospital, 30 Bond Street, Toronto, ON M5B 1W8 Canada; Department of Medicine, University of Toronto, Toronto, ON Canada

**Keywords:** HIV/AIDS, Disability, Questionnaire, Validity, Reliability

## Abstract

**Background:**

Our aim was to assess internal consistency reliability, construct validity, and test-retest reliability of the HDQ with adults living with HIV in Canada and Ireland.

**Methods:**

We recruited adults 18 years of age or older living with HIV from hospital clinics and AIDS service organizations in Canada and Ireland. We administered the HDQ paired with reference measures (World Health Organization Disability Assessment Schedule, SF-36 Questionnaire, Medical Outcomes Study Social Support Survey), and a demographic questionnaire. We calculated HDQ disability presence, severity and episodic scores (scored from 0–100). We calculated Cronbach’s alpha and Intraclass Correlation Coefficients (ICC) (Canada only) for the disability severity and episodic scores and considered coefficients >0.80 and >0.70 as acceptable, respectively. To assess construct validity, we tested 40 *a priori* hypotheses of correlations between scores on the HDQ and reference measures and two known group hypotheses comparing HDQ presence and severity scores based on age and comorbidity. We considered acceptance of at least 75 % of hypotheses as demonstrating support for construct validity.

**Results:**

Of the 235 participants (139 Canada; 96 Ireland), the majority were men (74 % Ireland; 82 % Canada) and were taking antiretroviral therapy (88 % Ireland; 91 % Canada). Compared with Irish participants, Canadian participants were older (median age: 48 versus 41 years) and reported living with a higher median number of comorbidities (4 versus 1). Cronbach’s alpha for Irish and Canadian participants were 0.97 (95 % confidence interval (CI): 0.97–0.98) and 0.96 (95 % CI: 0.95–0.98), respectively, for the severity scale and 0.98 (95 % CI: 0.97–0.98) and 0.96 (95 % CI: 0.95–0.98), respectively, for the episodic scale. Of the 40 construct validity correlation hypotheses, 32 (80 %) and 22 (55 %) were supported among the Canadian and Irish samples respectively; both (100 %) known group hypotheses were also supported. ICC values for Canadian participants ranged from 0.80 (95 % CI: 0.71, 0.86) in the cognitive domain to 0.89 (95 % CI: 0.83, 0.92) in the social inclusion domain.

**Conclusions:**

The HDQ demonstrates internal consistency reliability and a variable degree of construct validity when administered to adults living with HIV in Canada and Ireland. The HDQ demonstrates test-retest reliability when administered to adults with HIV in Canada. Further validation of the HDQ outside of Canada is needed.

**Electronic supplementary material:**

The online version of this article (doi:10.1186/s12955-015-0310-9) contains supplementary material, which is available to authorized users.

## Background

In developed countries such as Canada and Ireland where individuals living with HIV have access to treatment, HIV is increasingly experienced as a chronic illness. At the end of 2011, over 71,000 people were living with HIV in Canada and 7800 in Ireland [1, 2]. In both countries, the majority of new infections continue to include men who have sex with men (46 % Ireland; 47 % Canada) with 23–25 % of new diagnoses occurring among women [3]. As people with HIV in these countries are living longer, they are facing similar issues related to comorbidity and aging, such as bone and joint disorders, mental health conditions, cardiovascular disease, cancer, and neurocognitive decline [4–8]. Forming international partnerships in countries where individuals experience similar issues related to HIV and aging is essential to addressing clinical and research priorities in this emerging field.

The health-related challenges experienced with these comorbidities in addition to HIV and aging may be termed, *disability*. Disability is defined as a combination of physical, cognitive, mental and emotional symptoms and impairments; difficulties carrying out day-to-day activities; challenges to social inclusion; and uncertainty about future health that may be experienced as episodic in nature, fluctuating on a daily basis and over the extended course living with HIV [9].

Measuring disability in the context of HIV is important for determining the prevalence and impact of disability associated with HIV, comorbidities and aging, and for determining the effectiveness of interventions to reduce the presence and severity of disability. Dimensions of disability may overlap with other health-related concepts such health-related quality of life (HRQL) measured in the context of HIV. In an earlier content analysis we assessed the extent to which items in existing HIV-specific HRQL questionnaires captured the health-related challenges experienced by adults living with HIV according to the *Episodic Disability Framework*, a conceptual framework developed from the perspective of adults living with HIV [9, 10]. Many of the instruments included in this review were developed prior to the advent of combination antiretroviral therapy which dramatically changed the course of HIV infection from a progressively terminal illness to a chronic illness characterized by periods of wellness and illness [11]. Other recent developed HIV-specific HRQL questionnaires more broadly included items related to social relationships and employment [12, 13]. However these instruments were not developed to assess the episodic nature of disability; and components of HRQL such as personal goals, values and expectations go beyond the scope of disability. Furthermore, evidence demonstrating weak relationships between symptoms and function (domains of disability) and overall health-related quality of life suggest HRQL and disability while related, are distinct concepts, hence both are important to consider in the context of HIV [14, 15].

Originally developed in Canada, the HIV Disability Questionnaire (HDQ), is a self-administered instrument that describes the presence, severity and episodic nature of disability experienced by adults living with HIV [16]. Developed from the *Episodic Disability Framework*, the HDQ is the first known HIV-specific measure of disability developed and assessed for use with people living with HIV [9, 17]. Distinct features of the HDQ include its description of the daily episodic nature of disability, and inclusion of uncertainty as a domain of disability. The HDQ possesses sensibility, including face and content validity and ease of use for people living with HIV in Ontario [18]. However, the reliability and validity of the HDQ for use with people living with HIV in Canada and other developed countries is unknown.

Our aim was to assess the internal consistency reliability, construct validity, and test-retest reliability of the HDQ. We specifically focused on Ireland and Canada due to a funding opportunity to promote collaborative research between these two countries. Results from this study will help to establish the role of the HDQ as a future measure of disability in countries such as Canada and Ireland where HIV is considered a chronic illness and individuals experience similar issues related to HIV, comorbidity and aging.

## Methods

We conducted two cross-sectional studies to assess the measurement properties of the HDQ in Canada and Ireland. All aspects of this research were conducted in collaboration with a Community Advisory Committee based in Canada comprised of four members including adults living with HIV, representatives from AIDS Service Organizations and a representative from the Ontario Ministry of Health and Long-Term Care. This research was approved by Research Ethics Boards at McMaster University, Hamilton, Ontario, University of Toronto, St. Michael’s Hospital, Toronto, Ontario, Canada, and St. James’s Hospital, Dublin, Ireland.

### Participants and recruitment

We included adults 18 years of age or older living with HIV who self-identified as having experienced an episode of illness attributed to HIV and who were able to read and understand English. We recruited participants in southern Ontario, Canada and Dublin, Ireland from hospital clinics, HIV community-based service organizations and a specialty hospital (Casey House).

### Data collection

We administered the HDQ with seven additional instruments: the World Health Organization Disability Assessment Schedule 2.0 (WHODAS-2.0), SF-36 Health Questionnaire (SF-36), Centers for Epidemiologic Studies Depression Scale (CES-D), Medical Outcomes Study Social Support Survey (MOS-SSS), HIV Symptom Index, Brief COPE, and HIV Stigma Scale [19–25]. We also administered a demographic questionnaire. We administered the HIV Disability Questionnaire (HDQ) a second time one week later to the participants in Ontario to assess test-retest reliability.

#### HIV disability questionnaire

The HDQ consists of 69 items that describe the presence, severity and episodic nature of disability experienced by adults living with HIV. The HDQ is comprised of six domains: physical, cognitive and, mental and emotional health symptoms and impairments, uncertainty, difficulty with day-to-day activities and challenges to social inclusion [26]. An additional item asks participants to classify living with HIV as a ‘good day’ or ‘bad day’. Each item consists of a statement about a health-related challenge and has both a five point ordinal response scale asking the respondent to rate the challenge on the day of administration (from 0 to 4) and a nominal response scale asking whether the challenge fluctuated (or changed) over the past week (‘Yes’ or ‘No’). *Disability presence scores* are calculated by summing the number of health challenges experienced for each HDQ domain and transforming them to a score out of 100. *Disability severity scores* are calculated by summing individual item scores from each domain and then linearly transforming them into HDQ domain severity scores out of 100. *Episodic scores* are calculated by summing the number of challenges identified as fluctuating in each domain and then transforming them to a score out of 100. All HDQ scores range from 0–100. Higher scores indicate a greater presence, severity, and episodic nature of disability.

#### World Health Organization Disability Assessment Schedule 2.0 (WHODAS-2.0)

The WHODAS-2.0 contains 36 items that measures disability across six domains (cognition, mobility, self-care, getting along, life activities, and participation). Scores ranges from 0 to 100, where 0 means no disability and 100 means full disability [19]. The WHO-DAS 2.0 has been used with people living with HIV [27] and possesses reliability, validity and responsiveness among people with chronic disease [28].

#### Brief COPE

The Brief COPE contains 28 items about coping responses to stressful events living with a health condition [21]. Brief COPE scoring is divided into an Adaptive and Maladaptive Coping Score. The Adaptive Coping Score is the sum of 16 positive coping items related to active coping, planning, emotional support, instrumental support, positive reframing, acceptance, religion and humor (ranging from 16–64) with higher scores indicating better coping skills, and the Maladaptive Coping Score is the sum of 12 negative coping items related to venting, denial, substance use, behavioral disengagement, self-distraction and self-blame (ranging from 12–48) with higher scores indicating worse coping skills [21].

#### HIV stigma scale

The HIV Stigma Scale contains 40 items which ask about social and emotional aspects of having HIV [20]. The HIV Stigma Scale includes four subscales: personal stigma, disclosure, negative self-image and public attitudes (scores range from 40–160). Higher scores indicate higher levels of stigma [20]. The HIV Stigma Scale possesses construct validity and reliability with people living with HIV [20].

#### Medical Outcomes Study (MOS) social support scale

The MOS-Social Support Survey contains 19 items that ask how often companionship, assistance and other types of support are available if needed [24]. The MOS-Social Support Score ranges from 0 to 100, where 0 means no support available when needed, and 100 means support is always available when needed [24]. The MOS-Social Support Scale possesses construct validity and reliability when used with people living with HIV [24].

#### HIV symptom index

The HIV Symptom Index is a 20-item questionnaire that describes the presence and burdensome nature of symptoms experienced by adults with HIV. Each item is scored using a five point ordinal scale ranging from ‘I do not have this symptom’ to ‘I have this symptom and it bothers me a lot’. HIV Symptom Index scoring involves summing the number of symptoms that are present and the number that are considered bothersome. The HIV Symptom Index demonstrates good construct validity with people living with HIV [22].

#### CES-D

The Centers for Epidemiologic Studies for Depression Scale (CES-D) is a 20-item instrument that measures depressive symptomatology [23]. The Scale possesses high internal consistency, adequate test-retest reliability and concurrent and construct validity in the general population [23] and has been used extensively with people living with HIV [29–33]. Item scores range from 0–60 and higher scores indicate more depressive symptoms [23].

#### SF-36 questionnaire

The SF-36 questionnaire is a generic health-related quality of life instrument [25, 34]. The measurement properties of the SF-36 are well established, demonstrating high internal consistency reliability, content, construct, and predictive validity [35, 36]. Domain scores for the SF-36 include physical function, role limitation physical, bodily pain, vitality, role limitation emotional, mental health, social function, and general health perception. Physical Health Component Summary (PCS) and Mental Health Component Summary (PCS) scores are calculated from the domain scores. All domain and summary scores range on a scale from 0–100 with higher scores indicating better quality of life.

### Analysis

All data were entered into a database and 20 % of cases were independently manually verified for accuracy. We computed missing response rates for the disability and episodic sections of the HDQ and the health status measures. We removed cases with more than 10 % missing item responses in the HDQ. For all other cases with missing responses, we performed mean imputation.

We assessed the distribution of HDQ item responses and domain scores, and health status summary scores. We classified floor and ceiling effects as >15 % of responses being at the bottom or top of the scales, respectively [37]. We assessed differences between Canadian and Irish participants for HDQ and health status measures using the Mann-Whitney test for continuous variables and chi-square test for categorical variables (statistical significance *p* < 0.05). Statistical analyses were conducted using SAS and SPSS statistical software [38, 39].

### Internal consistency reliability

We calculated Cronbach’s alpha for the HDQ severity and episodic domain and total scores to assess internal consistency reliability, the degree to which the items within the instrument are correlated with each other. We considered Cronbach’s alpha >0.80 as acceptable [40].

### Construct validity

We tested 40 *a priori* construct validity hypotheses about predicted relationships using correlations between HDQ severity scores and three health status measures (WHO-DAS 2.0; SF36 questionnaire; MOS-Social Support Scale) and two hypotheses comparing HDQ presence and severity scores based on age and comorbidity. Specifically, we assessed 15 convergent hypotheses with the WHO-DAS 2.0 questionnaire, 18 convergent hypotheses with the SF-36 questionnaire, and seven divergent hypotheses with the MOS-Social Support Scale. We also tested two known group hypotheses (greater presence and severity of disability with age and number of comorbidities) (Additional file 1). Correlation coefficients of | ≥ 0.30|, | ≥ 0.50| and | ≥ 0.70|, were defined as ‘weak’ , ‘moderate, ’ and ‘strong, ’ respectively [41]. Each construct validity hypothesis and its corresponding hypothesized level of correlation are provided in Additional file 1. Fisher transformation was used to normalize the distribution and stabilize the variance, and to compute the confidence intervals [42]. We considered acceptance of at least 75 % of hypotheses determined by the correlation coefficient as demonstrating support for construct validity (Additional file 1) [43].

### Test-retest reliability

We assessed test-retest reliability by calculating an intra-class coefficient (ICC) with Canadian participants who completed the HDQ at time 1 and time 2, one week later. We included participants who did not report a major change in their health between time points and who reported no change in the ‘good day/bad day’ classification item. We considered an ICC > 0.70 as acceptable for test-retest reliability [40, 44].

#### Sample size estimation

Sample size estimation was based on the validity analysis and our desire to detect a weak correlation between the scores of the criterion reference measures and the HDQ scores. To detect a Pearson’s correlation coefficient of *r* = 0.30 with a power of 0.90, and an alpha of 0.05, we required at least 113 participants [45] for each study. To adjust for an estimated 15 % of questionnaires with missing responses and loss to follow-up at time 2 for the test-retest reliability analyses, we required at least 130 participants. Using a low correlation coefficient was a conservative approach to sample size estimation.

## Results

We recruited 142 participants in Ontario between April-July 2012 and 101 participants in Dublin between June-July 2012, of whom 139 and 100 participants were eligible and agreed to participate, respectively. Four people from Dublin completed less than half of the questionnaires and were removed from the dataset, resulting in 139 Canadian, and 96 Irish participants in the study (Table 1). The majority of Irish participants (89; 93 %) were recruited from St. James’s Hospital GUIDE Clinic in Dublin, whereas the majority of Canadian participants (129; 91 %) were recruited from HIV community based service organizations in southern Ontario.

**Table 1:** Characteristics of participants (*n* = 235)

Characteristic	Canada (*n* = 139)	Ireland (*n* = 96)	*P* value
	Number (%)	Number (%)	
*Gender*			0.149
Men	114 (82 %)	72 (74 %)	
Women	24 (17 %)	23 (24 %)	
Other	1 (1 %)	2 (2 %)	
Age (median; 1^st^–3^rd^ quartile)	48 years (44–55 years)	41 years (34–48 years)	<.001*
50 years or older	58 (41 %)	22 (23 %)	
Year of Diagnosis (median: 1^st^-3^rd^ quartile)	1999 (1990–2004)	2003 (1998–2009)	<.001*
Diagnosed with HIV prior to 1996	58 (42 %)	13 (14 %)	<.001*
Taking antiretroviral therapy	127 (91 %)	84 (88 %)	0.457
Undetectable Viral Load^a^	110 (89 %)	41 (85 %)	0.463
Currently working for pay	29 (21 %)	52 (54 %)	<.001*
Living alone	91 (66 %)	28 (29 %)	<.001*
Have children	36 (26 %)	33 (34 %)	<.001*
Live with children	11 (8 %)	24 (25 %)	
*Self*-*rated health status*			<.001*
Poor	12 (9 %)	3 (3 %)	
Fair	35 (25 %)	10 (10 %)	
Good	56 (40 %)	21 (22 %)	
Very good	25 (18 %)	34 (35 %)	
Excellent	11 (8 %)	26 (27 %)	
Number of concurrent health conditions (median; 1^st^–3^rd^ quartile)	4 (2–6)	1 (0–3)	<.001*
*Common concurrent health conditions*			
Muscle pain	77 (56 %)	21 (22 %)	<.001*
Mental health	65 (47 %)	18 (19 %)	<.001*
Joint pain	60 (44 %)	22 (23 %)	0.002*
Addiction	43 (31 %)	9 (9 %)	<.001*
Neurocognitive decline	43 (31 %)	11 (12 %)	<.001*
Hepatitis C	17 (12 %)	21 (22 %)	0.047*
High blood pressure	33 (24 %)	16 (17 %)	0.207

Not all characteristics add to the total n due to missing responses

*Statistically significant difference (*p* < 0.05) between groups of participants

^a^out of 123 and 48 responses from Canadian and Irish participants, respectively

### Characteristics of participants

Of the 235 participants (139 Canada; 96 Ireland), the majority were men (74 % Ireland; 82 % Canada) taking antiretroviral therapy (88 % Ireland; 91 % Canada) with an undetectable viral load (85 % Ireland; 89 % Canada) (Table 1). Compared with Irish participants, Canadian participants were older (median age: 48 versus 41 years), living longer with their HIV diagnosis (median year of diagnosis 1999 in Canada; 2003 in Ireland), reported living with a higher number of comorbidities (4 in Canada versus 1 in Ireland), tended to live alone (66 % in Canada versus 29 % in Ireland), and were less frequently working for pay (21 % in Canada versus 54 % in Ireland). Canadian participants tended to rate their health status as either fair or good whereas Irish participants tended to rate their health status as very good or excellent (Table 1).

#### Health status questionnaires

Compared to Irish participants, Canadians had higher WHO-DAS 2.0 disability scores, lower mental and physical health summary scores on the SF-36 questionnaire (indicating lower quality of life), higher CES-D scores (indicating higher levels of anxiety and depression), higher HIV Symptom Index Scores (indicating more symptoms), higher maladaptive and adaptive coping scores on the Brief COPE, and lower scores on the MOS-Social Support Scale (indicating less social support) (all *p* < 0.05). Scores were similar between groups on the HIV Stigma Scale (Table 2).

**Table 2:** Characteristics of participants: health status measures

Measure	Canada (*n* = 139)	Ireland (*n* = 96)	*P* value
	Median (IQR)	Median (IQR)	
World Health Organization Disability Assessment Schedule (WHODAS-2.0)			
(Range 0–100)	30 (18,44)	12 (5,24)	<.001*
SF-36 questionnaire (Range 0–100)			
Mental component summary score	39 (32,49)	47 (38,54)	0.001*
Physical component summary score	43 (35,50)	53 (43,57)	<.001*
CES-D summary score			
(Range 0–60)	23 (15,33)	13 (6,21)	<.001*
HIV symptom index (Range 0–20)			
Total # present	16 (11,19)	11 (5,15)	<.001*
Total # bothersome	13 (8,16)	7 (3,11)	<.001*
HIV stigma scale (Range 40–160)	103 (84,117)	99 (86,118)	0.756
MOS-social support scale			
(Range 1–100)	49 (29,74)	63 (43,89)	0.001*
Brief COPE			
Adaptive (Range 16–64)	42 (36,48)	37 (30,45)	0.002*
Maladaptive (Range 12–48)	22 (19,28)	20 (16,24)	0.001*

*Statistically significant difference between groups (*p* < 0.05)

### HIV disability questionnaire

Canadian and Irish participants took a median of 10 min (1st-3rd quartile: 8–12 min) and 13 min (1^st^–3^rd^ quartile: 10–15 min) to complete the HDQ, respectively. Missing item responses were <3 % across all HDQ disability and episodic items. We observed a ceiling effect (>15 % of responses indicating the highest severity of disability) for eight items with Canadian participants and four items with Irish participants. Ceiling effects were most common for items pertaining to uncertainty or worrying about the future. All 69 HDQ items demonstrated a floor effect (>15 % of responses indicating no disability) for Canadian and Irish participants. Of these, 28 and 54 HDQ items had >40 % of responses indicating no disability for the Canadian and Irish participants, respectively. Floor effects were most common for items that referred to symptoms and impairments or difficulties with day-to-day activities.

Median HDQ presence, severity and episodic scores are displayed in Table 3. Highest median disability presence scores were in the cognitive (Canada: 100) and uncertainty domains (Ireland: 71). Highest median disability severity scores were in the uncertainty domain for both Canada (score: 39) and Ireland (score: 30). The median episodic score, representing the number of challenges that fluctuated in the past week, was highest in the physical symptoms and impairments domain and similar between samples (20 Ireland and Canada). Median HDQ severity and presence scores were significantly higher among Canadian participants across all domain and total scores, except for uncertainty for which they were similar (Table 3).

**Table 3:** Median HIV Disability Questionnaire (HDQ)scores (*n* = 235)

Disability dimension	HDQ Presence Score (IQR)			HDQ Severity Score (IQR)			HDQ Episodic Score (IQR)		
	Canada	Ireland	*P* value	Canada	Ireland	*P* value	Canada	Ireland	*P* value
Physical	60 (40–60)	35 (15,60)	0.0001*	25 (11, 38)	13 (5,25)	0.0001*	20 (5,55)	20 (0,40)	0.226
Cognitive	100 (33, 100)	33 (0100)	0.0001*	25 (17, 42)	8 (0,25)	0.0001*	0 (0,67)	0 (0,33)	0.167
Mental-emotional	73 (45, 91)	45 (18,80)	0.0001*	30 (13, 50)	14 (7,30)	0.0001*	9 (0,45)	9 (0,36)	0.615
Uncertainty	79 (57, 93)	71 (50,93)	0.073	39 (23, 61)	30 (18,53)	0.086	0 (0,29)	0 (0,36)	0.936
Difficulties with day-to-day activities	56 (22, 89)	11 (0,22)	0.0001*	17 (6, 31)	3 (0,8)	0.0001*	0 (0,22)	0 (0,0)	0.004*
Challenges to social inclusion	67 (50, 92)	42 (19,58)	0.0001*	31 (17, 50)	17 (7,29)	0.0001*	0 (0,17)	0 (0,8)	0.383
HDQ total	70 (43, 81)	43 (26,59)	0.0001*	29 (16, 42)	17 (8,26)	0.0001*	12 (1,39)	12 (3,28)	0.523

*Statistically significant difference between Canadian and Irish participants (Mann-Whitney Test) (*p* < 0.05)

Episodic HDQ scores were similar between groups. The most common episodic health challenges included fatigue (52 % Canada; 38 % Ireland), followed by feeling sad, down or depressed (44 % Canada; 35 % Ireland), aches and pains (37 % Canada and Ireland), nausea (39 % Canada; 20 % Ireland), shortness of breath (36 % Canada; 28 % Ireland), and feeling anxious (35 % Canada; 32 % Ireland). Eighty-one percent (time 1) and 82 % (time 2) of Canadian participants and 88 % of Irish participants completed the HDQ on what they considered a ‘good day’ living with HIV.

### Validity and reliability

#### Internal consistency reliability

All HDQ severity and episodic domain and total scores met pre-specified criteria for internal consistency reliability. Cronbach’s alpha for the HDQ severity domain scores ranged from 0.84 (95 % confidence interval (CI): 0.76, 0.92) in the cognitive domain to 0.93 (95 % CI: 0.91, 0.95) in the mental-emotional domains for the Irish and Canadian participants respectively (Table 4). Cronbach’s alpha for the HDQ episodic scores ranged from 0.81 in the cognitive domain to 0.95 in the uncertainty domain (Table 4).

**Table 4:** Internal consistency reliability of the HIV Disability Questionnaire (HDQ)

HDQ score	HDQ severity score		HDQ episodic score	
	Cronbach’s alpha (95 % CI)		Cronbach’ alpha (95 % CI)	
	Canada	Ireland	Canada	Ireland
Physical	0.92 (0.90, 0.94)	0.89 (0.86,0.92)	0.92 (0.91 , 0.94)	0.88 (0.84,0.92)
Cognitive	0.87 (0.82, 0.91)	0.84 (0.77,0.90)	0.81 (0.74, 0.88)	0.84 (0.76,0.92)
Mental-emotional	0.93 (0.91, 0.95)	0.91 (0.88,0.94)	0.91 (0.89, 0.94)	0.90 (0.87,0.94)
Uncertainty	0.93 (0.91, 0.94)	0.92 (0.90,0.94)	0.95 (0.94, 0.97)	0.94 (0.92,0.97)
Difficulty with day-to-day activities	0.91 (0.83, 0.93)	0.88 (0.83,0.94)	0.92 (0.89, 0.95)	0.85 (0.77,0.93)
Challenges to social inclusion	0.90 (0.88, 0.93)	0.90 (0.85,0.94)	0.94 (0.92, 0.97	0.90 (0.85,0.94)
HDQ total (all items)	0.97 (0.97, 0.98)	0.96 (0.95,0.98)	0.98 (0.97, 0.98)	0.96 (0.95,0.98)

Interpretation: Cronbach alpha > 0.80 defined as acceptable

*CI* Confidence Interval

#### Construct validity

Of the 40 construct validity correlation hypotheses, 80 and 55 % were supported by Canadian and Irish participants respectively and both known groups hypotheses (100 %) were confirmed (Table 5). Of the correlation HDQ hypotheses, 87 and 67 % that referred to the WHO-DAS scores, 78 and 67 % that referred to the SF36 scores, and 71 and 0 % that referred to the MOS-Social Support Scale scores were supported by Canadian and Irish participants respectively. For details on each of the 42 construct validity hypotheses and resulting correlation coefficients see Additional file 1. Bolded correlation coefficients and 95 % confidence intervals indicate a given hypothesis was confirmed (Additional file 1).

**Table 5:** Summary of construct validity analysis results

Hypotheses tested	# of Hypotheses tested	# of Hypotheses confirmed (%)	
		Canada	Ireland
Convergent construct validity	15	13 (87 %)	10 (67 %)
WHODAS 2.0 scores will moderately(≥0.50) to strongly (≥0.70) correlatewith HDQ scores			
Convergent construct validity	18	14 (78 %)	12 (67 %)
SF-36 scores will weakly (≥0.30) to strongly (≥0.70) correlate with HDQ scores			
Divergent construct validity	7	(71 %)	0 (0 %)
MOS-SS scores will weakly (≥0.30) to moderately (≥0.50) correlate with HDQ scores			
Total # of correlation hypotheses tested	40	32 (80 %)	22 (55 %)
Known groups validity	2	2 (100 %)	
Older participants with morecomorbidity (Canadian sample) will have higher total HDQ presence and severity scores			

See Additional file 1 for details of all 42 hypotheses and the correlation coefficients

#### Test-retest reliability

Ninety-nine Canadian participants reported having no major change in their health and reported no change in the good day/bad day item on the HDQ and were included in the test-retest reliability analysis. Results demonstrated that the HDQ domain and total scores were stable over time with ICC values ranging from 0.80 (95 % CI: 0.71, 0.86) in the cognitive domain to 0.89 (95 % CI: 0.83, 0.92) in the challenges to social inclusion domain (Table 6).

**Table 6:** Internal consistency reliability of the HIV Disability Questionnaire (HDQ) (*n* = 99)

HDQ domain	Intraclass Correlation Coefficient (ICC) (95 % CI)
	(*n* = 99)
Physical	0.83 (0.64, 0.91)
Cognitive	0.80 (0.71, 0.86)
Mental-emotional	0.88 (0.80, 0.93)
Uncertainty	0.85 (0.78, 0.90)
Difficulty with day-to-day activities	0.86 (0.80, 0.90)
Challenges to social inclusion	0.89 (0.83, 0.92)
HDQ total	0.90 (0.83, 0.94)

Interpretation: ICC >0.70 considered acceptable

Includes Canadian participants with no major change in health and no change in good day/bad day item (*n* = 99)

## Discussion

The HDQ demonstrated internal consistency reliability and construct validity when administered to adults living with HIV in Canada and Ireland. This is the first known HIV-specific measure developed to assess the presence, severity and episodic nature of disability among adults with HIV developed from a conceptual model of disability [9]. This study builds on previous evidence that established the domain and scoring structure of the HDQ and confirmed the validity of the six domain structure with adults living with HIV in Ontario [26, 46].

Internal consistency reliability assessment resulted in Cronbach’s alpha >0.80 for all severity and episodic domain and total scores, suggesting the items in the HDQ are homogeneous within the six HDQ domains to collectively measure the broader construct of disability at one point in time. Test-retest reliability results among the Canadian participants indicated ICC ≥0.80 for all HDQ domain and total scores suggesting the HDQ is consistent at measuring disability over time.

Pre-specified construct validity criteria were confirmed for 80 % (Canada) and 55 % (Ireland) of hypothesized relationships between the HDQ and health reference measures and both known group hypotheses. Thus, our results indicate that the HDQ satisfied construct validity (>75 % of hypotheses) for Canadian, but not for Irish, participants. Differences in construct validity between Canada and Ireland may be attributed to the mechanism in which the HDQ and reference measures were administered. For example, Canadian participants completed the measures consecutively in one single sitting in a quiet location at an HIV service organization, whereas Irish participants completed the measures intermittently while seeing various health providers in a busy HIV clinic. This may have introduced inconsistencies in the way participants responded to items across the questionnaires resulting in lower correlations between measures. For instance, if a participant attended clinic with a level of disability, the HDQ scores (administered prior to the health provider encounter) may be higher or lower compared with SF36 questionnaire scores (administered post encounter) depending on whether the disability was influenced by the event of seeing the health provider. Differences in construct validity also may be attributed to differences in culture, and the willingness of participants to disclose their vulnerability to chronic illness and the health-related challenges of HIV [47]. Cross-cultural adaptation of the HDQ may be considered when administering the instrument in other countries with varying culture or perceptions related to chronic illness [48, 49]. Finally, lower levels of confirmed hypotheses were found among the seven *a priori* hypotheses for divergent construct validity with the MOS-Social Support Scale in both samples (71 % Canada; 0 % Ireland) suggesting we may have overestimated the extent to which we hypothesized items in the HDQ would correlate with items of social support.

Notably Canadian participants were older, living longer with their HIV diagnosis, reported a greater number of comorbidities, tended to live alone, and fewer were working for pay in some capacity (full time, part time, or under the table). These differences were reflected in the HDQ and health status measures with Canadian participants reporting higher disability, lower quality of life, higher depression, more symptoms, and lower levels of social support compared with Irish participants. Differences between the samples were likely attributed to the different mechanism of recruitment across countries. The majority of the Irish sample (93 %) was recruited from a hospital clinic that broadly served adults living with HIV receiving care in Dublin, whereas the majority of the Canadian sample was primarily recruited from HIV community-based organizations (91 %), or a specialty hospital (7 %) whereby participants likely were experiencing health-related challenges requiring them to seek out additional social support or health services. Nevertheless, floor effects (scores closer to the lower end of the scale) were evident across the HDQ in both samples and to a greater extent among Irish participants. This may have decreased the ability for Irish HDQ scores to correlate with the other measures that possessed more normalized distributions of scores. Floor and ceiling effects can pose challenges for responsiveness and diminish the ability for an instrument to detect a change if one occurs [50]. Removing items that demonstrate a large floor or ceiling effect, or adding items at the lower end of the scale, may be considered in future HDQ revision to increase the distribution of scores and enhance the ability to discriminate between participants.

Higher coping scores among the Canadian participants suggest that they engaged in both adaptive and maladaptive coping strategies more frequently compared with Irish participants. The higher frequency of coping may be attributed to the greater comorbidity and severity of disability experienced by Canadian participants which may result in their adopting both forms of coping (adaptive and/or maladaptive) more frequently. Alternatively, Irish participants who were living with less comorbidity and disability may not feel the need to adopt these strategies to the same extent.

Unlike all other HDQ domains whereby Canadian participants reported more disability, uncertainty was the highest HDQ domain severity score and it was similar among both Canadian and Irish participants. The concept of uncertainty is particularly relevant to aging with HIV, whereby older adults may worry about their source of health challenges; health providers’ knowledge and skills; financial uncertainty; transition to retirement; appropriate long-term housing and who will care for them as they age living with HIV [51].

Compared with other HDQ domains, physical symptoms and impairments tended to fluctuate more on a daily basis with median HDQ episodic scores greatest in the physical symptoms domain (20 challenges fluctuated within the week among Irish and Canadian samples) followed by mental emotional symptoms and impairments (nine challenges), demonstrating the potential episodic nature of disability. Items related to symptoms and impairments such as fatigue, weakness and trouble concentrating may fluctuate more readily than those associated with social inclusion such as the ability to engage or re-engage in the workforce [52]. Specific symptoms and impairments that fluctuated the most included fatigue, feeling sad, down or depressed, nausea, aches and pains, shortness of breath, and feeling anxious. The episodic scale of the HDQ represents the daily episodic nature of disability rather than the larger fluctuations in health that may occur over a longer period of time. Further work is needed to assess the properties of the HDQ episodic scale.

The majority of participants (81–88 %) reported completing the HDQ on a ‘good day’ living with HIV despite the presence and severity of disability reported in the HDQ. This may be a reflection of resiliency, adaptation and hardiness among adults aging with HIV as a chronic disease [53, 54]. Nevertheless, it is unclear how participants defined a ‘good day’ versus a ‘bad day’ living with HIV. Further work exploring the interpretation of this item as it relates to the HDQ disability scores is needed.

### Implications for practice, research and policy

The HDQ may be used by clinicians and HIV community organizations to assess disability experienced by their clients as a result of HIV and other concurrent health conditions. This may help to identify areas of need where programs, services and interventions that reduce disability experienced by clients with HIV can be effective. Further, given the findings suggest insufficient validity in Ireland, further refinement and validity assessment of the HDQ beyond Canada is needed. Future revision of the HDQ also may include a short-form version to enhance the feasibility of the HDQ for use in the clinical setting. Future assessment of interpretability will be critical to understand the meaning of HDQ scores for adults living with HIV and clinicians. Additional psychometric assessment of responsiveness will enable researchers to use the HDQ to document changes in disability over time and determine the effectiveness of interventions.

Our study has limitations. First, the HDQ was developed with men living with HIV living in a large metropolitan city who were taking antiretroviral therapy, living with concurrent health conditions, and not currently working. Accordingly, the HDQ demonstrated higher construct validity among Canadian participants who resembled the sample from which the HDQ was originally derived, validated and refined. While this study was the first to assess the validity of the HDQ internationally, the properties of the HDQ among people living with HIV in the developing country context are unknown. Second, because our goal was to assess the measurement properties of the HDQ rather than to measure disability per se, the HDQ scores should be considered cautiously since the interpretability of the HDQ scores are unknown. Lastly, our analysis focused on assessing the construct validity, internal consistency reliability and test-retest reliability of the domains of the disability severity scale of the HDQ. Test–retest reliability assessment was limited to Canadian participants; hence further investigation of this property outside of Canada is needed.

## Conclusions

The HDQ demonstrates internal consistency reliability and a variable degree of construct validity when administered to adults living with HIV in Canada and Ireland. The HDQ demonstrates test-retest reliability when administered to adults with HIV in Canada. Differences in construct validity between countries may be due to lower HDQ scores among Irish participants who were younger and reported less comorbidity, and differences in the mechanism of questionnaire administration. Further work to explore HDQ applications outside of Canada is needed.

## Additional file

Additional file 1:
**Details of construct validity analysis.** (DOCX 229 kb)

## Abbreviations

CES-D: Centers for Epidemiologic Studies Depression Scale (CES-D)
CI: Confidence Interval
HIV: Human Immunodeficiency Virus
HDQ: HIV Disability Questionnaire
HRQL: Health-related quality of life
MOS-SS: Medical Outcomes Study Social Support Survey
WHODAS-2.0: World Health Organization Disability Assessment Schedule 2.0
