# Supplementary material for: Reliability and validity of a new HIV-specific questionnaire with adults living with HIV in Canada and Ireland: the HIV Disability Questionnaire (HDQ)
Source: Health Qual Life Outcomes. 2015 Aug 12;13:124. doi: 10.1186/s12955-015-0310-9 (PMC4542093; doi:10.1186/s12955-015-0310-9)
Supplement: Additional file 1: — Details of construct validity analysis. (DOCX 229 kb) [file 12955_2015_310_MOESM1_ESM.pdf]

Additional File 1: Details of Construct Validity Analysis

**Construct Validity Analysis**	**Pearson Correlation Coefficient (r)** (95% confidence interval)	
**Convergent Construct Validity Hypotheses with the WHODAS 2.0 (15 hypotheses)**	**Canada (n=139)**	**Ireland (n=96)**
1) Total WHODAS scores will strongly correlate (≥0.70) with HDQ total scores	**0.89 (0.85, 0.92)**	**0.81 (0.72, 0.87)**
2) Cognition WHODAS summary scores will strongly correlate (≥0.70) with the cognitive domain scores of the HDQ	**0.74 (0.66, 0.81)**	**0.73 (0.62, 0.81)**
3) Cognition WHODAS summary scores will moderately correlate (≥0.50) with the mental-emotional domain scores of the HDQ	**0.61 (0.49, 0.70)**	**0.63 (0.50, 0.74)**
4) Mobility WHODAS summary scores will strongly correlate (≥0.70) with the difficulties with day-to-day domain scores of the HDQ	**0.81 (0.74, 0.86)**	**0.75 (0.64, 0.83)**
5) Self-Care WHODAS summary scores will strongly correlate (≥0.70) with the difficulties with day-to-day domain scores of the HDQ	**0.71 (0.62, 0.78)**	0.43 (0.25, 0.58)
6) Getting Along WHODAS summary scores will moderately correlate (≥0.50) with the social inclusion domain scores of the HDQ	**0.73 (0.64, 0.80)**	**0.63 (0.49, 0.74)**
7) Life-Activities (Household) WHODAS summary scores will strongly correlate (≥0.70) with the difficulties with day-to-day domain scores of the HDQ	0.66 (0.56, 0.74)	0.64 (0.51, 0.75)
8) Life-Activities (Work) WHODAS summary scores will moderately correlate (≥0.50) with the social inclusion domain scores on the HDQ	**0.67 (0.55, 0.76)**	**0.51 (0.33, 0.65)**
9) Participation WHODAS summary scores will strongly correlate (≥0.70) with the social inclusion domain scores on the HDQ	**0.80 (0.73, 0.85)**	**0.79 (0.70, 0.86)**
10) Total WHODAS scores will strongly correlate (≥0.7) with the physical symptoms and impairments domain scores of the HDQ	**0.73 (0.64, 0.80)**	0.58 (0.43, 0.70)
11) Total WHODAS scores will strongly correlate (≥0.7) with the cognitive symptoms and impairments domain scores of the HDQ	**0.71 (0.62, 0.79)**	0.64 (0.50, 0.74)
12) Total WHODAS scores will strongly correlate (≥0.7) with the mental-emotional symptoms and impairments domain scores of the HDQ	**0.73 (0.64, 0.80)**	**0.70 (0.58, 0.79)^**

13) Total WHODAS scores will strongly correlate (≥0.7) with the difficulties with day-to-day activities domain scores of the HDQ.	**0.78 (0.70, 0.84)**	0.69 (0.57, 0.78)^
14) Total WHODAS scores will strongly correlate (≥0.7) with the challenges to social inclusion domain scores of the HDQ	**0.80 (0.73, 0.85)**	**0.76 (0.66, 0.83)**
15) Total WHODAS scores will moderately correlate (≥0.5) with the uncertainty domain scores of the HDQ	**0.69 (0.60, 0.77)^**	**0.56 (0.41, 0.69)**
**Total Number of Hypotheses Confirmed**	**13/15 (87%)**	**10/15 (67%)**
**Convergent Construct Validity Hypotheses with the SF-36 Questionnaire (18 hypotheses)**	**Canada (n=139)**	**Ireland (n=96)**
1) PCS Scores on the SF36 will moderately correlate (≥0.5)with the physical symptoms and impairments domain scores of the HDQ	**-0.63 (-0.72, -0.52)**	**-0.68 (-0.78, -0.56)**
2) MCS Score on the SF36 will moderately correlate (≥0.5)with the mental – emotional symptoms and impairments domain scores of the HDQ	-0.73 (-0.80, -0.64)	-0.70 (-0.79, -0.58)^
3) Bodily Pain Score on the SF36 will strongly correlate (≥0.7) with the physical symptoms and impairments domain scores of the HDQ	**-0.70 (-0.78, -0.60)^**	-0.67 (-0.77, -0.54)
4) Vitality Summary Score on the SF36 will strongly correlate (≥0.7) with the physical symptoms and impairments domain scores of the HDQ	-0.61 (-0.71, -0.50)	-0.63 (-0.74, -0.49)
5) Social Function Summary Score on the SF36 will moderately correlate (>0.5) with the physical symptoms and impairments domain scores of the HDQ	**-0.57 (-0.67, -0.44)**	**-0.56 (-0.68, -0.41)**
6) Physical Function score on the SF36 will moderately correlate (≥0.5) with the difficulties with day-to-day activities domain scores of the HDQ	**-0.67 (-0.75, -0.56)**	**-0.63 (-0.74, -0.49)**
7) Role Physical Summary Score on the SF36 will moderately correlate (≥0.5) with the difficulties with day-to-day activities domain scores of the HDQ	**-0.60 (-0.70, -0.49)**	**-0.63 (-0.74, -0.49)**
8) Role Physical Summary Score will moderately correlate (≥0.5) with the social inclusion domain scores of the HDQ	**-0.60 (-0.70, -0.48)**	**-0.56 (-0.69, -0.41)**
9) Role Physical Summary Score will moderately correlate (≥0.5) with the physical domain scores of the HDQ	**-0.62 (-0.71, -0.50)**	**-0.56 (-0.68, -0.40)**

10) Role Emotional Summary Score will moderately correlate (>0.5) with the mental-emotional domain scores of the HDQ	**-0.63 (-0.72, -0.52)**	-0.48 (-0.62, -0.31)
11) Role Emotional Summary Score will moderately correlate (>0.5) with the social domain scores of the HDQ	**-0.61 (-0.71, -0.50)**	**-0.54 (-0.66, -0.38)**
12) Mental Health Summary Score will strongly correlated (>0.7) with the mental-emotional domain scores of the HDQ	**-0.76 (-0.82, -0.68)**	-0.68 (-0.78, -0.56)
13) Social Function Summary Score will moderately correlate (>0.5) with the mental-emotional domain scores of the HDQ	**-0.64 (-0.72, -0.52)**	**-0.67 (-0.77, -0.54)**
14) Social Function Summary Score will moderately correlate (>0.5) with the social domain scores of the HDQ	**-0.60 (-0.70, -0.48)**	**-0.65 (-0.75, -0.52)**
15) Social Function Summary Score will moderately correlate (>0.5) with the physical domain scores of the HDQ	**-0.57 (-0.67, -0.44)**	**-0.56 (-0.68, -0.41)**
16) Mental Component Summary (MCS) Score will moderately correlate (>0.5) to with the uncertainty domain scores of the HDQ	-0.73 (-0.80, -0.64)~	-0.47 (-0.61, -0.30)
17) Physical Component Summary (PCS) Score will weakly correlate (>0.3) with the uncertainty domain scores of the HDQ	**-0.31 (-0.46, -0.15)**	**-0.34 (-0.50, -0.15)**
18) Mental Component Summary (MCS) Score will moderately correlate (>0.5) with the cognitive domain scores of the HDQ	-0.47 (-0.59, -0.32)	**-0.57 (-0.69, -0.42)**
**Total Number of Hypotheses Confirmed**	**14/18 (78%)**	**12/18 (67%)**
**Divergent Construct Validity with MOS-Social Support Scale (7 hypotheses)**	**Canada (n=139)**	**Ireland (n=96)**
1) Scores on the Social Support Scale will weakly correlate (>0.3) with the physical domain scores of the HDQ	**-0.34 (-0.48, -0.18)**	-0.12 (-0.32, 0.08) p=0.22
2) Scores on the Social Support Scale will weakly correlate (>0.3) with the cognitive domain scores of the HDQ	-0.29 (-0.44, -0.13)^	-0.11 (-0.3, 0.09) p=0.29
3) Scores on the Social Support Scale will moderately correlate (>0.5) with the mental-emotional domain scores of the HDQ	**-0.52 (-0.63, -0.38)**	-0.33 (-0.50, -0.14)

4) Scores on the Social Support Scale will weakly correlate (>0.3) with the UNCERTAINTY domain of the HDQ	**-0.48 (-0.60, -0.33)**	-0.17 (-0.36, 0.03) p=0.10
5) Scores on the Social Support Scale will weakly correlate (>0.3) with the DAY domain of the HDQ	-0.26 (-0.41, -0.10)	-0.24 (-0.42, -0.04)
6) Scores on the Social Support Scale will moderately correlate (>0.5) with the SOCIAL domain of the HDQ	**-0.56 (-0.67, -0.43)**	-0.34 (-0.51, -0.15)
7) Scores on the Social Support Scale will weakly correlate (>0.3) with the HDQ TOTAL Score	**-0.49 (-0.62, -0.36)^**	-0.27 (-0.45, -0.08)
**Total Number of Divergent Construct Validity Hypotheses Confirmed**	**5/7 (71%)**	**0/7 (0%)**
**Total Number of Correlation Hypotheses Confirmed**	**32/40 (80%)**	**22/40 (55%)**
**Known Groups Construct Validity (2 hypotheses)**		
1) Older participants with more comorbidity will have higher total HDQ presence scores	**Confirmed** (Canada: 70 versus Ireland: 43) (p = 0.0001)	
2) Older participants with more comorbidity will have higher total HDQ severity scores	**Confirmed** (Canada: 29 versus Ireland: 17) (p = 0.0001)	
**Total Number of Known Group Hypotheses Confirmed**	**2/2 (100%)**	

LEGEND
All correlation coefficients were statistically significant (p<0.0001) unless otherwise stated.
Bolded coefficients indicate acceptance of (or confirmed) hypotheses.
^Indicate seven borderline correlation coefficients values of weak (|0.3|), moderate (|0.5|) and strong (|0.7|) correlations
~Strongly correlated (|>=0.70|) rather than moderately correlated (|>=0.50|)
